# Is Salvage of a Kidney Graft Possible as a Result of Hyperacute Rejection Immediately After Kidney Transplantation?

**DOI:** 10.7759/cureus.50538

**Published:** 2023-12-14

**Authors:** Irshad Hussain, Salma Mahmoud, Scott Schurman, Reut Hod Dvorai, Rauf Shahbazov

**Affiliations:** 1 Internal Medicine/Nephrology, Upstate University Hospital, Syracuse, USA; 2 Surgery, Upstate University Hospital, Syracuse, USA; 3 Pediatric Nephrology, Upstate University Hospital, Syracuse, USA; 4 Pathology/Immunology, Upstate Medical University, Syracuse, USA; 5 Transplant Division, Department of Surgery, Albany Medical Center, Albany, USA

**Keywords:** graft survival, anti-hla antibodies, living donor, kidney transplantation, hyperacute rejection

## Abstract

Hyperacute rejection is a rare complication of renal transplantation. It is mainly caused by preformed human leukocyte antigen antibodies and can lead to the loss of the transplanted kidney. Renal transplantation is a highly beneficial treatment for people with end-stage renal disease, greatly improving their quality of life. However, antibody-mediated rejection is a significant challenge for the long-term survival of transplanted kidneys.

An 18-year-old male with nephrotic syndrome, who underwent bilateral renal nephrectomy due to severe proteinuria, received a living donor kidney. Pretransplant panel reactive antibodies were low. Cytotoxic T- and B-cell and non-HLA cross-match was negative. The graft became cyanotic and mottled within half an hour of transplantation. Allograft was quickly extracted, and a biopsy showed hyperacute rejection. The patient was treated with plasmapheresis, intravenous immunoglobulin, and eculizumab. The graft was successfully re-implanted after 18 hours. Further treatment included additional sessions of plasmapheresis, intravenous immunoglobulin, eculizumab, T-cell-depleting agent, and immunosuppressive therapy. Serum creatinine became stable, and renal biopsy after one month demonstrated intact parenchyma with no inflammation or fibrosis.

This case highlights the critical importance of promptly removing the transplanted kidney and using aggressive immunotherapy to save renal allografts in cases of hyperacute rejection.

## Introduction

Renal transplantation is a preferred treatment for end-stage renal disease and greatly enhances quality of life [1]. However, antibody-mediated rejection poses a significant challenge to long-term graft survival and can lead to graft dysfunction or loss [2]. Antibody-mediated rejection encompasses various types, including hyperacute, acute, and chronic rejection [3,4]. Hyperacute rejection, a rare occurrence in renal allograft transplantation, is typically triggered by preformed HLA antibodies. This can be prevented by conducting pretransplant T- and B-cell cross-matching [3,5-7].

Recent evidence suggests that endothelial/non-HLA antibodies, which are not routinely detected through standard cross-match methods, may also play a role in mediating rejection [7,8]. Despite negative results in prospective T- and B-cell cross-matching and the absence of endothelial/non-HLA antibodies, hyperacute rejection can still manifest. While management strategies such as plasmapheresis and high-dose intravenous immunoglobulin have shown efficacy in preventing and treating antibody-mediated rejection [9-11], the lack of a standardized therapeutic approach remains a significant concern regarding graft loss.

## Case presentation

An 18-year-old male, with a significant past medical history of focal segmental glomerulosclerosis, underwent bilateral native nephrectomy due to severe proteinuria. Subsequently, he received a living donor kidney transplant from his mother. The patient had a heterozygous *Nephrin *mutation, which was the underlying cause of focal segmental glomerulosclerosis. Two weeks before the transplant, the pretransplant calculated panel-reactive antibodies were 16%, which increased to 56% on the day of the transplant but subsequently decreased to less than 1%. The cytotoxic T- and B-cell cross-match results were negative, and the patient did not develop donor-specific anti-HLA antibodies.

**Figure 1:**
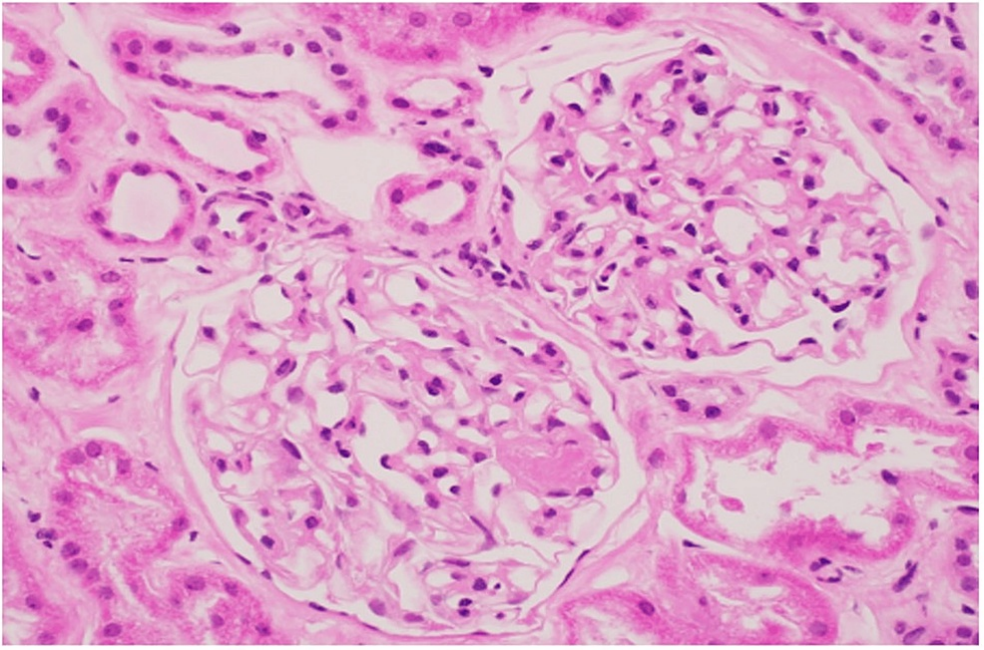
Formalin-fixed permanent section of a biopsy with confirmation of neutrophil infiltration and glomerular capillary microthrombus (hematoxylin and eosin stain at 20× original magnification).

**Figure 2:**
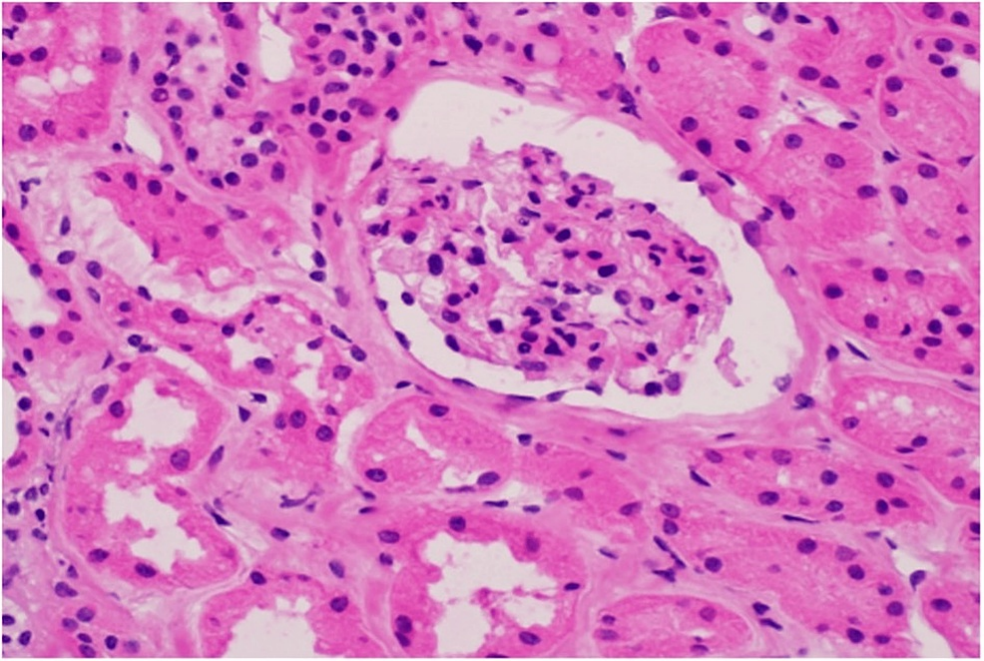
Neutrophils accumulating in glomerular capillaries in the frozen section of a biopsy taken in the operating room shortly after implantation of the allograft (hematoxylin and eosin stain at 40× original magnification).

The initial perfusion of the transplanted kidney appeared satisfactory, but within 30 minutes, the graft displayed cyanosis and a mottled appearance despite exhibiting good intra-graft Doppler ultrasound signals. The transplant kidney was promptly devascularized, extracted, and flushed with the University of Wisconsin solution. A frozen section of a wedge biopsy revealed the presence of neutrophilic glomerulitis and small arteriolar thrombi, consistent with hyperacute rejection (Figures 1, 2).

The allograft was subjected to hypothermic machine perfusion overnight, and the pump parameters indicated favorable results (flow: 110 mL/minute, RI: 0.24). To address the rejection, the patient received treatment comprising plasmapheresis, intravenous immunoglobulin (400 mg/kg), and eculizumab (1,200 mg) to inhibit complement activation. After 18 hours, the allograft was successfully reimplanted. Following the surgery, the patient underwent five sessions of plasmapheresis every other day, along with administration of eculizumab (600 mg), intravenous immunoglobulin (200 mg/kg), and rabbit anti-thymocyte globulin (total dose of 7.5 mg/kg). The patient’s immunosuppression regimen included prednisone, mycophenolate mofetil, and tacrolimus.

**Figure 3:**
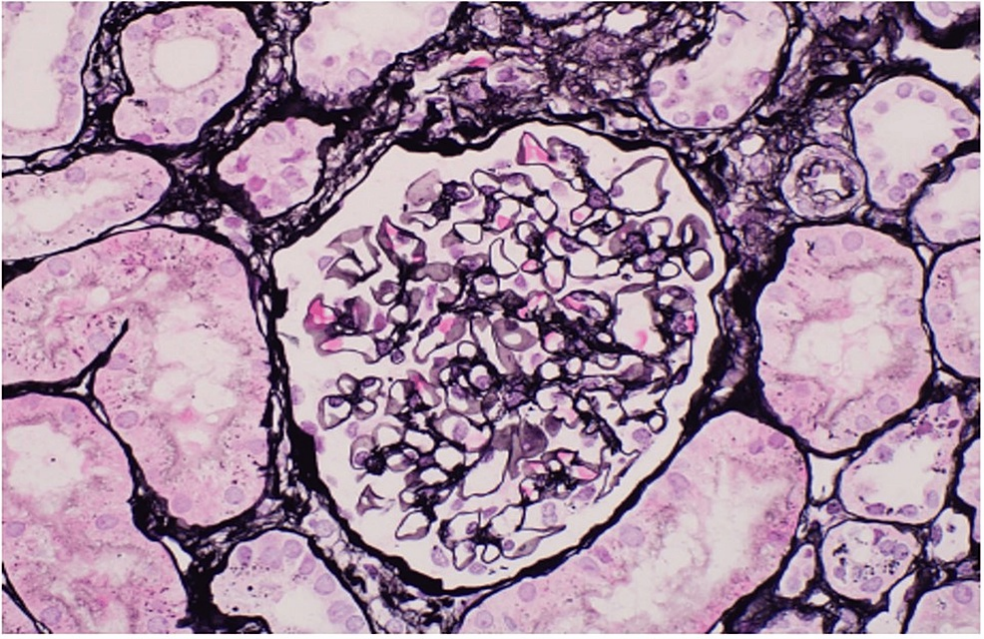
Normal glomeruli and tubules on follow-up biopsy (Jones silver at 40× original magnification).

Serum creatinine levels steadily improved, reaching below 4 mg/dL within one week and stabilizing at 2.0-2.3 mg/dL at three months. A repeat allograft biopsy conducted at one month indicated intact parenchyma without any signs of inflammation or fibrosis, and C4d staining in peritubular capillaries was negative (Figure 3). A retrospective T- and B-cell flow cross-match performed on a sample from the day of the transplant yielded negative results. Additionally, endothelial cross-match and non-HLA antibody testing, including angiotensin II type 1 receptor and major histocompatibility complex class I-related chain A, showed negative results. Genetic screening for complement regulatory mutations did not provide a diagnostic outcome.

## Discussion

The occurrence of hyperacute rejection is an unusual event in this modern era due to worldwide pretransplant cross-matching to detect pre-existing donor-specific antibodies. Non-HLA antibodies have been reported as responsible in the absence of HLA antibodies [12], and they can be identified through non-HLA antibody testing before allograft transplantation. Histopathology typically reveals arteritis, interstitial edema, and severe cortical necrosis, which may necessitate transplant nephrectomy [3].

Therapeutic approaches involve antibody depletion through plasmapheresis; immunomodulation with intravenous immunoglobulin; T-cell depletion using anti-thymocyte globulin; immunosuppression with mycophenolate mofetil, tacrolimus, and prednisone; and targeting the terminal complement pathway with eculizumab. No definitive treatment exists, and combinations of therapies are employed to optimize hyperacute rejection, particularly in highly sensitized patients before transplantation.

This case illustrates that conventional pretransplant cross-matching may not always prevent hyperacute rejection; however, graft salvage is possible through immediate graft extraction and aggressive immunotherapy, as outlined above. The use of eculizumab in combination with conventional antibody-mediated rejection therapy can prevent early graft loss [13,14], but randomized studies are necessary to assess its extensive utilization. Nonetheless, the high cost limits its use. Vaccination against meningococcus and pneumococcus is mandatory before initiating eculizumab.

Despite extensive investigation in our case, the etiology remained unidentified. While advanced techniques aid in detecting HLA and non-HLA donor-specific antibodies, alloimmune response continues to pose a significant challenge [13]. Further research is needed to enhance the efficacy of pharmacotherapy and gain a better understanding of the pathophysiology, diagnosis, and treatment of hyperacute rejection.

## Conclusions

In this specific case, an innovative strategy is introduced to address hyperacute rejection, a condition that is not commonly encountered and currently lacks any established methods of treatment. This novel approach holds significant promise in effectively addressing the unique challenges presented by hyperacute rejection. The swift removal of the transplanted organ, along with intensive immunotherapy and the application of eculizumab, showed positive results in effectively managing this condition. The progress observed in this specific case serves as evidence for the favorable outcomes. To verify the effectiveness and potential implications, it is crucial to conduct thorough investigations and further research.

## References

[1] Tonelli M, Wiebe N, Knoll G (2011). Systematic review: kidney transplantation compared with dialysis in clinically relevant outcomes. Am J Transplant.

[2] Davis S, Cooper JE (2017). Acute antibody-mediated rejection in kidney transplant recipients. Transplant Rev (Orlando).

[3] Puttarajappa C, Shapiro R, Tan HP (2012). Antibody-mediated rejection in kidney transplantation: a review. J Transplant.

[4] Nankivell BJ, Alexander SI (2010). Rejection of the kidney allograft. N Engl J Med.

[5] Patel R, Terasaki PI (1969). Significance of the positive crossmatch test in kidney transplantation. N Engl J Med.

[6] Pereira M, Guerra J, Gonçalves J, Santana A, Nascimento C, da Costa AG (2016). Hyperacute rejection in a kidney transplant with negative crossmatch: a case report. Transplant Proc.

[7] Tittelbach-Helmrich D, Bausch D, Drognitz O (2014). Hyperacute rejection of a living unrelated kidney graft. Case Rep Med.

[8] Sumitran-Karuppan S, Tyden G, Reinholt F, Berg U, Moller E (1997). Hyperacute rejections of two consecutive renal allografts and early loss of the third transplant caused by non-HLA antibodies specific for endothelial cells. Transpl Immunol.

[9] Wan SS, Ying TD, Wyburn K, Roberts DM, Wyld M, Chadban SJ (2018). The treatment of antibody-mediated rejection in kidney transplantation: an updated systematic review and meta-analysis. Transplantation.

[10] Montgomery RA, Loupy A, Segev DL (2018). Antibody-mediated rejection: new approaches in prevention and management. Am J Transplant.

[11] Cooper JE (2020). Evaluation and treatment of acute rejection in kidney allografts. Clin J Am Soc Nephrol.

[12] McCaughan JA, Tinckam KJ (2018). Donor specific HLA antibodies & allograft injury: mechanisms, methods of detection, manifestations and management. Transpl Int.

[13] Garces JC, Giusti S, Staffeld-Coit C, Bohorquez H, Cohen AJ, Loss GE (2017). Antibody-mediated rejection: a review. Ochsner J.

[14] Tan EK, Bentall A, Dean PG, Shaheen MF, Stegall MD, Schinstock CA (2019). Use of eculizumab for active antibody-mediated rejection that occurs early post-kidney transplantation: a consecutive series of 15 cases. Transplantation.

